# 14-3-3ζ-A Novel Immunogen Promotes Inflammatory Cytokine Production

**DOI:** 10.3389/fimmu.2019.01553

**Published:** 2019-07-24

**Authors:** Jenna McGowan, Cara Peter, Saurabh Chattopadhyay, Ritu Chakravarti

**Affiliations:** ^1^Department of Physiology and Pharmacology, University of Toledo College of Medicine and Life Sciences, Toledo, OH, United States; ^2^Department of Medical Microbiology and Immunology, University of Toledo College of Medicine and Life Sciences, Toledo, OH, United States

**Keywords:** 14-3-3ζ, autoantigen, T cell polarization, Interferon-gamma, IL-17, HLA-DRB1

## Abstract

The presence of autoantibodies against 14-3-3ζ in human autoimmune diseases indicates its antigenic function. However, neither the cause nor the consequence of this newly-identified antigenic function of 14-3-3ζ protein is known. To address this, we investigated the immunological functions of 14-3-3ζ by studying *ex vivo* effects on human peripheral blood mononuclear cells (PBMC) proliferation, polarization, and cytokine production. Exogenous 14-3-3ζ promoted PBMC proliferation and T cell polarization toward Th1 and Th17 populations. Significant increases in IFN-γ and IL-17 levels were observed in the presence of 14-3-3ζ. A specific increase in Th1 cells and IFN-γ production provided strong evidence for MHC class II presentation of 14-3-3ζ antigen. Particularly HLA-DRB1^*^0401 allele strongly promoted 14-3-3ζ-induced IFN-γ producing cells. In contrast, prednisolone treatment suppressed both 14-3-3ζ-induced T cell polarization and cytokine production. Overall, we show that MHC presentation and the adaptor functions of 14-3-3ζ participate in promoting IFN-γ and IL-17 production, two of the cytokines commonly associated with autoimmune diseases. To the best of our knowledge, this is the first report describing the *ex vivo* antigenic function of 14-3-3ζ with human PBMC, thereby providing the basis of its immunological role in human diseases.

## Introduction

Autoantigens are principal components of autoimmune diseases (1–3). Reactivity to a specific antigen is a key determinant of autoimmune and allergic responses, and often correlates with the clinical symptoms (4–6). The steps of internalization and processing of antigens for presentation to the MHC are mostly well characterized (7, 8). However, the influence of an antigen on the immune response is unique and complex (4, 9). The complexity of factors including HLA specificity, the presence of antigen-specific T and B cells, and the presence of multiple or overlapping epitopes, are a few of the challenges in the understanding of antigenicity (10). These factors contribute to the limited development of autoimmune disease therapy (9, 11). There is a need to increase our understanding of how antigens influence the innate immune response, and thereby contribute to the pathogenesis of diseases (9).

We recently reported 14-3-3ζ as a novel autoantigen in human large vessel vasculitis (LVV) patients with aortic aneurysms (12). The presence of 14-3-3ζ cleaved peptide in human sera is reported to regulate lymphocyte recruitment and is altered in rheumatoid arthritis (13). The antigenic function of 14-3-3ζ is also reported in several cancers, including hepatocellular carcinoma and small cell lung cancer (14–16). The antigenic function of other members of the 14-3-3 family *viz*. η, θ and ε proteins are also emerging in several human diseases (17–19). The isoform-specific sequences may be responsible for their unique antigenic functions (20). In addition to antigenic function, the increased extracellular presence of 14-3-3ζ and other isoforms is known in the context of tissue homeostasis and diseases (19, 21–25). Unlike cellular functions, such as signaling, cellular proliferation, migration, and stress-response (26–28), immune functions of 14-3-3 family members are recent and evolving. In order to understand the immunological functions of 14-3-3 proteins in human diseases, we need to characterize the antigenicity and elucidate the cellular basis of immune functions.

To gain insight into the role of 14-3-3ζ in autoimmune diseases, we decided to investigate the mechanism of its immunological functions. Our results show that 14-3-3ζ-induced T cell polarization favors Th1 and Th17 cells in human peripheral blood mononuclear cells (PBMC) *ex vivo* and has a significant role in promoting IFN-γ and IL-17A (henceforth, IL-17) production. Interestingly, 14-3-3ζ-induced IFN-γ, but not IL-17, production depends upon MHC class II presentation. The presence of autoimmune susceptible HLA-DRB^*^0401 allele led to a significant increase in 14-3-3ζ-induced IFN-γ production. On the other hand, the presence of a 14-3-3 competitive inhibitor suppressed cytokine production without significant impact on T cell polarization. Treatment with prednisolone can effectively inhibit Th17 and cytokine (IFN-γ and IL-17) production. Collectively, our results highlight a novel mechanism to the physiological role of 14-3-3ζ as an immunogen in promoting the inflammatory immune response.

## Results

### 14-3-3ζ Promotes Human PBMC Viability and Cytokine Secretion

**Figure 1:**
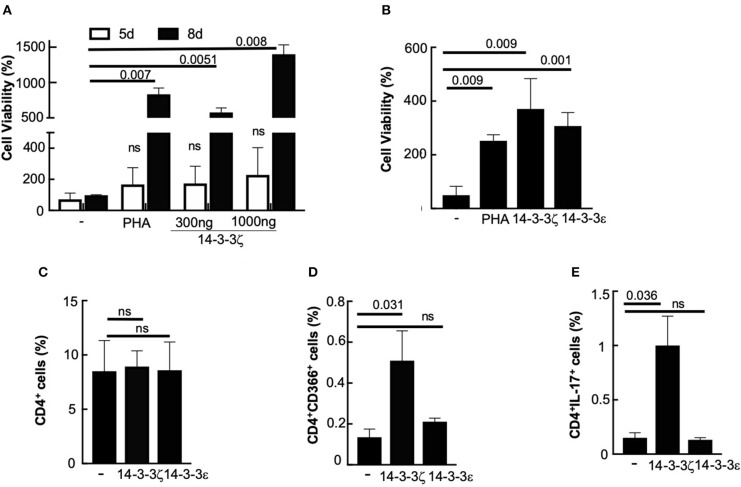
14-3-3ζ promotes human PBMC viability and T cell polarization. **(A,B)** The 50,000 PBMC per well (in triplicate) either from banked **(A)** or fresh **(B)** blood were tested for MTT assay after 8d (or 5d) post-incubation with purified 14-3-3ζ protein at 1,000 ng/ml (or 300 ng/ml). PHA (2%) was used as a positive control. **(C–E)** PBMC were incubated with 1 μg/ml of 14-3-3ζ for 7d were gated for CD4-FITC positive cells, and cells that co-stained for either CD366-APC or IL-17-Alexa Fluor 647 positive were quantitated. 14-3-3ε was used as isoform-control to test the specificity of 14-3-3ζ (*n* = 4, *p*-values are shown and ns, non-significant).

LVV is a disease that is dominated by T-cells and increased levels of IFN-γ and IL-17 (29). We discovered that 14-3-3ζ is an autoantigen in LVV (12), and therefore, hypothesized that the presence of 14-3-3ζ dictates T cell polarization and cytokine levels. To test this, we analyzed the effect of recombinant 14-3-3ζ on PBMC viability and T cell polarization. First, we measured the proliferative response of human PBMC to 14-3-3ζ, which exhibited a dose-dependent increase in PBMC viability (Figure 1A). Phytohemagglutinin (PHA), a known mitogen, served as a positive control in our MTT assay (Figures 1A,B). It was interesting to observe that PBMC isolated from banked blood, in comparison to fresh blood, showed a stronger response to 14-3-3ζ. To avoid any effect of additives, we studied PBMC isolated from fresh blood obtained from healthy volunteers (Figure 1A vs. Figure 1B). Incubation with 14-3-3ζ also protected PBMC from the cell death (20 vs. 32%), as a lesser number of PI-stained cells were observed upon treatment (Supplementary Figure 1). Next, we investigated the effect of 14-3-3ζ on the number of total CD4^+^, Th1 (CD4^+^CD366/TIM-3^+^), and Th17 (CD4^+^IL-17^+^) cells, using our gating strategy as shown in Supplementary Figure 2B (more details in Supplementary Figures 2, 3; Supplementary Table 1). Incubating PBMC with 14-3-3ζ had no significant effect on the number of total CD4^+^ cells (Figure 1C); however, a significant increase in Th1 (CD4^+^CD366^+^), as well as Th17 (CD4+IL-17+) cells, was observed (Figures 1D,E). No significant effects of 14-3-3ζ on total CD8^+^, or activated CD8^+^ cells or the Treg population were observed (Supplementary Figures 4A–C; Supplementary Table 1). We further examined the specificity of this phenomenon by testing another isoform of 14-3-3. In contrast to 14-3-3ζ, treatment with 14-3-3ε had no significant effect on either Th1 or Th17 cells (Figures 1B–E).

**Figure 2:**
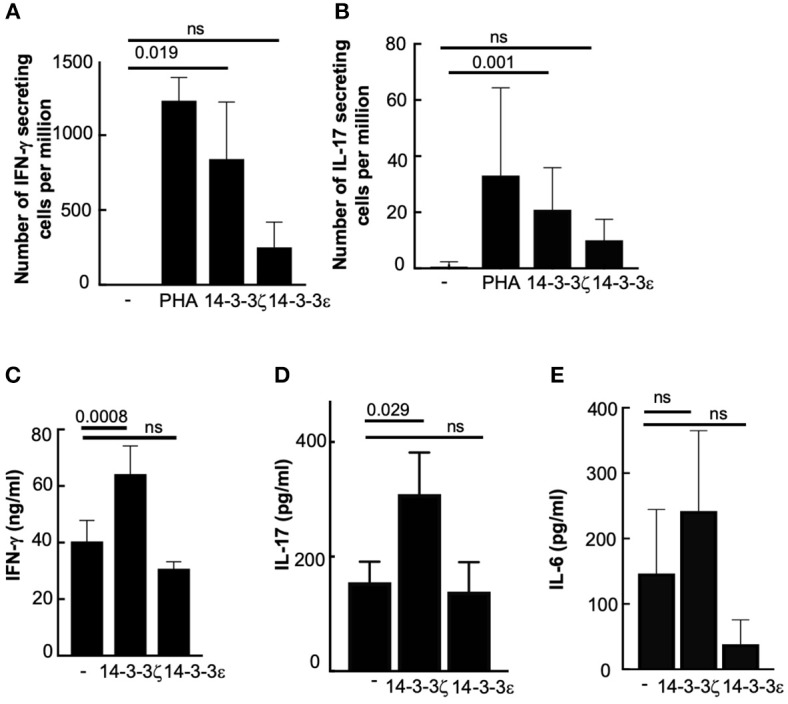
14-3-3ζ promotes IFN-γ and IL-17 production from human PBMC. **(A,B)** PBMC treated with 1 μg/ml of 14-3-3ζ for 7d were tested for a total number of IFN-γ **(A)**, or IL-17 **(B)**, producing cells using 100,000 cells per well of ELISPOT assay. Both PHA and 14-3-3ε were utilized as controls. **(C–E)** Accumulated IFN-γ **(C)**, IL-17 **(D)**, and IL-6 **(E)** in the conditioned media of PBMC treated with 14-3-3ζ for 7d were measured by ELISA. 14-3-3ε was used as a comparison and to demonstrate specificity (*n* > 3, *p*-values are shown, and ns, non-significant).

To test whether 14-3-3ζ-induced T cell polarization leads to cytokine secretion, we examined the effect of 14-3-3ζ on the number of cytokine-secreting cells and accumulated cytokines in the culture media of treated PBMC. Treatment with 14-3-3ζ resulted in a robust increase in the number of IFN-γ and IL-17 secreting cells, analyzed by ELISPOT assay (Figures 2A,B; Supplementary Figure 5). Importantly, this effect was specific to 14-3-3ζ, as 14-3-3ε had no significant effect on the cytokine-secreting cells (Figures 2A,B). Expectedly, PHA acted as positive control and promoted the number of cells secreting IFN-γ and IL-17 (Figures 2A,B). In contrast, no effect of 14-3-3ζ on the IL-10 or IL-12 secreting cells was observed (Supplementary Figures 4D,E). The increased numbers of IFN-γ and IL-17 producing cells led to higher levels of both cytokines in the culture media of 14-3-3ζ-treated PBMC (Figures 2C,D). Though statistically insignificant, an increased amount of IL-6 was secreted by the 14-3-3ζ-treated PBMC (Figure 2E). Also, IL-2 supplementation to amplify the proliferative response of PBMC and CD69 staining was found ineffective (Supplementary Figures 6A,B).

### MHC Class II Participates in 14-3-3ζ Antigenic Function

The antigenic properties of proteins require their association and presentation by specific HLA alleles (30). Because such information is not available for 14-3-3ζ, we first utilized a pan HLA antibody that binds to a common epitope of the beta subunit of HLA-DR (and HLA-DP) and examined the effect on 14-3-3ζ-induced cytokine-producing cells. The presence of anti-HLA antibody primarily suppressed the 14-3-3ζ-stimulated increase in the IFN-γ producing cells, but had no significant effect on the number of IL-17 producing cells. In contrast, the presence of anti-14-3-3ζ antibody during incubation suppressed the induction of both IFN-γ and IL-17 producing cells (Supplementary Figure 7).

**Figure 3:**
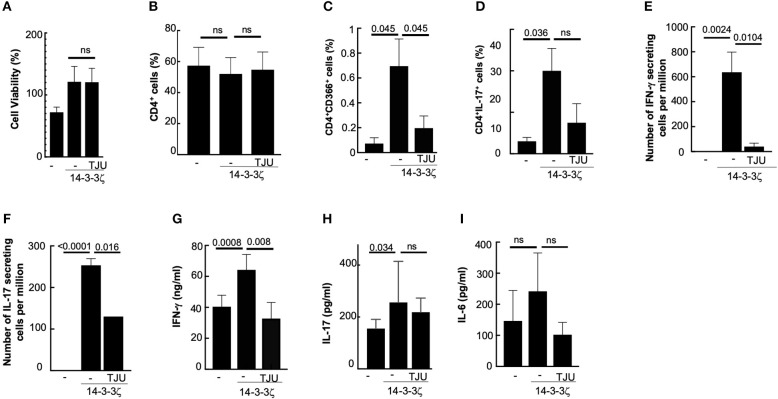
MHC class II participates in 14-3-3ζ-induced immune response. **(A)** Cellular viability of 50,000 PBMC per well (in triplicate) after 7d treatment with 1 μg/ml of 14-3-3ζ and TJU103 is shown. **(B–D)** Effect of TJU103 on the PBMC during 14-3-3ζ incubation was measured by testing CD4 positive cells **(B)**, that co-stained for either CD366 **(C)**, or IL-17 **(D)**, by FACS analysis. **(E,F)** Effect of TJU103 on IFN-γ **(E)** and IL-17 **(F)** producing cells was measured by co-incubating cells for 7d with 14-3-3ζ and inhibitor followed by plating cells for ELISPOT assay. **(G–I)** Accumulated cytokines in the conditioned media at the end of 7d incubation of PBMC with antigen and TJU103 were measured using ELISA (*n* = 3, *p*-values are shown, and ns, non-significant).

The involvement of HLA-DR4, activation of CD4^+^ cells, and the absence of activated CD8^+^ cells led us to hypothesize that MHC class II presentation participates in the antigenic function of 14-3-3ζ. To further test this, we used a small molecule inhibitor, TJU103, to block CD4-MHC class II interaction (31). We tested the effect of TJU103 on T cell polarization and cytokine production. PBMC in the presence of TJU103 during the 14-3-3ζ incubation did not change cell viability (Figure 3A). Blocking the CD4-MHC class II interaction did not affect the total percentages of CD4^+^ cells (Figure 3B); however, a stronger suppression in the number of Th1 cells and IFN-γ producing cells was observed (Figures 3C,E). Contrary to IFN-γ, IL-17 producing cells were less sensitive to the TJU103 treatment (Figures 3D,F). The effect on cytokine producing cells translated into corresponding cytokine levels in the culture media with significant inhibition of IFN-γ while no difference in the accumulated IL-6 or IL-17, respectively, when TJU103 was present (Figures 3G–I).

### Role of HLA-DRB1^*^04 on 14-3-3ζ Antigenic Function

**Figure 4:**
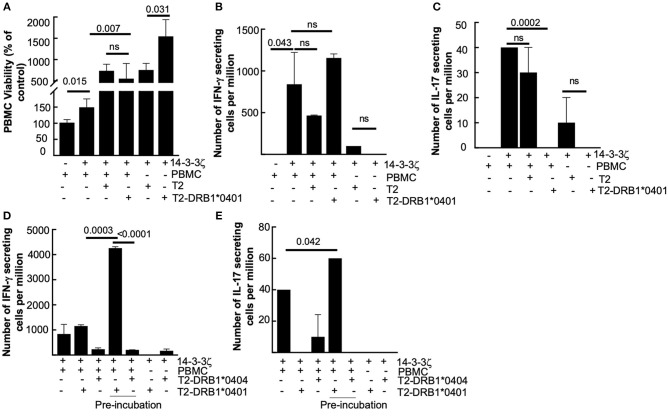
DRB1*0401 allele strongly upregulates 14-3-3ζ-induced IFN-γ, but is not immunodominant. **(A)** Effect of T2 (MHC class II deficient-empty) or DRB1*0401 expressing cells on PBMC viability was studied. **(B,C)** T2 cells expressing DRB1*0401 or control vector were incubated with PBMC (1:10 ratio) and 1 μg/ml of 14-3-3ζ for 7d followed by ELIPSOT assay to study the effect on IFN-γ **(B)** and IL-17 **(C)** producing cells. The number of spots per million PBMC are indicated. **(D,E)** T2 cells carrying either DRB*0401 or DRB1*0404 allele were incubated with 14-3-3ζ for 24 h followed by washes to remove the unprocessed antigen. Washed T2 cells were then cultured with PBMC (in 1:10 ratio) for 7d followed by ELISPOT assay to measure the total number of IFN-γ **(D)** and IL-17 **(E)** producing cells (*n* = 3, *p*-values are shown, and ns, non-significant).

Certain HLA alleles such as HLA-DRB1^*^0401 show associations with autoimmune diseases, including rheumatoid arthritis and vasculitis (32, 33). Encouraging results of HLA involvement in 14-3-3ζ-stimulated increase in IFN-γ secreting cells led us to further examine the effect of specific HLA-DRB1^*^04 alleles on the antigenic function of 14-3-3ζ. Here, we utilized specialized T2 (class II-deficient) cells co-expressing either vector control or HLA-DRB1^*^0401 (with HLA-DM) (34) to test their effect on 14-3-3ζ-induced cytokine production. We studied the effect on cell proliferation by incubating T2 or T2-DRB^*^0401 cells with PBMC (in a ratio of 1 T2 cell: 10 PBMC) in the presence of 14-3-3ζ. The presence of T2 cells resulted in a further increase in viable PBMC in HLA independent manner, but no significant effect on the number of IFN-γ producing cells was observed (Figures 4A,B). However, T2 cells expressing DRB1^*^0401 allele suppressed the number of IL-17 producing cells (Figure 4C). As expected, incubation of T2 or T2-DRB1^*^0401 cells with 14-3-3ζ, in the absence of PBMC, did not induce IFN-γ or IL-17 (last two columns of Figures 4B,C). This result made us question if HLA-DRB1^*^0401 allele can associate and present 14-3-3ζ to influence PBMC cytokine production. To test this, we incubated T2-HLA-DRB^*^0401 cells with 14-3-3ζ for 24 h, followed by multiple washes to remove the unprocessed protein. The T2 cells expressing HLA-DRB1^*^0404 allele were used as control. The freshly isolated PBMC were then incubated with pretreated and washed T2 cells expressing a specific HLA allele (in a ratio of 1 T2 cell: 10 PBMC) for 7 d followed by ELISPOT assay to examine the cytokine-producing cells. Pretreated T2 cells expressing HLA-DRB^*^0401 generated a much stronger induction of IFN-γ production, while a modest increase was observed in the case of IL-17 producers (Figures 4D,E). In contrast, T2 cells expressing DRB1^*^0404 allele did not promote any cytokine production (Figures 4D,E). These results suggest that 14-3-3ζ presentation by HLA-DRB1^*^0401 induces strong IFN-γ production.

### Prednisolone Suppresses 14-3-3ζ-Induced Cytokine Production

**Figure 5:**
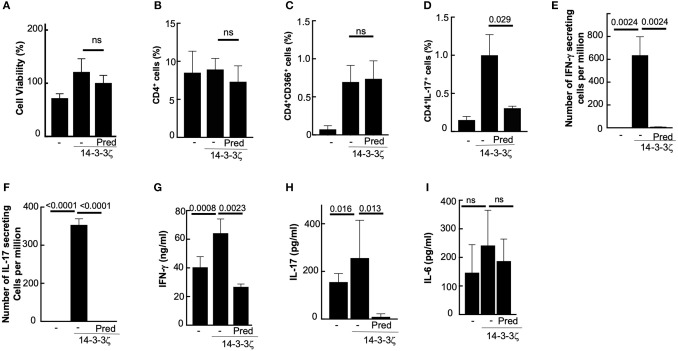
Prednisolone suppresses 14-3-3ζ induced T cell polarization and cytokine secretion. **(A)** Effect of 1 μg/ml prednisolone (Pred) on the PBMC viability was measured using MTT assay after 7d of treatment with 14-3-3ζ. **(B–D)** Effects of Pred on the CD4^+^ cells **(B)**, CD4^+^CD366^+^, **(C)** and CD4^+^IL-17^+^ **(D)** cells in the PBMC after 7d of 14-3-3ζ incubation was measured by FACS. **(E,F)** Number of IFN-γ **(E)** and IL-17 **(F)** producing cells in the PBMC incubated with 14-3-3ζ in the presence of Pred were counted using the ELISPOT assay. **(G–I)** Accumulated IFN-γ **(G)** IL-17 **(H)** and IL-6 **(I)** in conditioned media by PBMC at the end of 7d treatment with antigen and drug treatment were quantitated by ELISA (*n* = 3, *p*-values are shown, and ns, non-significant).

Glucocorticosteroids are widely used to suppress inflammation and control the clinical symptoms in autoimmune diseases (35, 36). Some of these drugs are known for differential effects on Th1 and Th17 cells, while others have global effects (37). We studied the 14-3-3ζ–induced cell proliferation in response to immunosuppressive prednisone (clinical drug) and prednisolone (active component). The presence of prednisone did not affect 14-3-3ζ-induced T cell polarization, cytokine secreting cells, or accumulated cytokines (data not shown). In contrast, prednisolone, in spite of no significant effects on cellular viability and the total percentage of CD4^+^ cells (Figures 5A,B), had significant suppression of Th17 cells (Figure 5D), IL-17 secreting cells (Figure 5F), and accumulated IL-17 levels (Figure 5H). Prednisolone, although, had no significant effect on Th1 cells (Figure 5C), exhibited a significant reduction in the total number of IFN-γ secreting cells (Figure 5E) and the level of secreted IFN-γ (Figure 5G). Expectedly, prednisolone treatment did not affect 14-3-3ζ-induced IL-6 secretion (Figure 5I).

### Contribution of 14-3-3ζ Adaptor Function to the Cytokine Production

**Figure 6:**
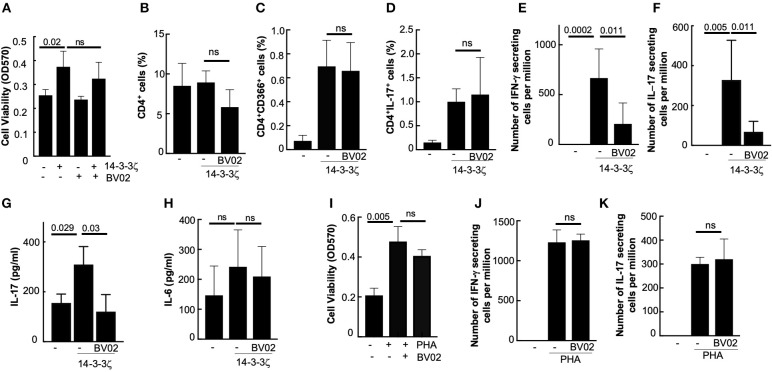
14-3-3 inhibitor BV02 suppresses cytokine production. **(A)** Effect of BV02 on the cellular viability of PBMC treated with 14-3-3ζ for 7d was measured by MTT assay. **(B–D)** Effect of BV02 on the 14-3-3ζ-induced effect on the total number of CD4^+^ **(B)**, CD4^+^CD366^+^ **(C)**, and CD4^+^IL-17^+^ **(D)** cells in PBMC was studied. **(E,F)** Effect of BV02 on the number of cells secreting IFN-γ **(E)** and IL-17 **(F)** that were induced by 14-3-3ζ was measured in the ELISPOT assay. **(G,H)** Accumulated IL-17 **(G)** and IL-6 levels **(H)** in conditioned media after 7d of 14-3-3ζ treatment of PBMC were measured by ELISA. **(I)** Effect of BV02 on the PBMC viability after the 7d treatment with PHA was measured by MTT assay. **(J,K)** Effect of BV02 on the PHA-induced increase in the number of IFN-γ **(J)** and IL-17 **(K)** secreting cells was measured by ELISPOT assay (*n* > 3, *p*-values are shown, and ns, non-significant).

Because 14-3-3 proteins have additional cellular functions involving interactions with other proteins, we tested if inhibiting these functions would affect 14-3-3ζ's antigenic functions, i.e., PBMC viability, T cell polarization, and cytokine production. A small molecule inhibitor (BV02), which prevents 14-3-3ζ interactions with other proteins (38), did not affect the 14-3-3ζ-induced PBMC viability or T cell polarization (Figures 6A–D). However, BV02 treatment of PBMC resulted in stronger suppression of the 14-3-3ζ-induced increase in the cytokine secreting cells (both IFN-γ and IL-17) as well as the accumulated IL-17 levels (Figures 6E–G). However, no significant effect of BV02 on IL-6 was observed (Figure 6H). These results suggest that unlike T cell polarization, the cytokine production by exogenous 14-3-3ζ requires cellular adaptor functions. As expected, PHA-induced PBMC proliferation and cytokine secretion, which are independent of 14-3-3, were unaffected by BV02 (Figures 6I–K). This shows that endogenous 14-3-3 does not participate in the cytokine production in the case of PHA stimulus.

## Discussion

**Figure 7:**
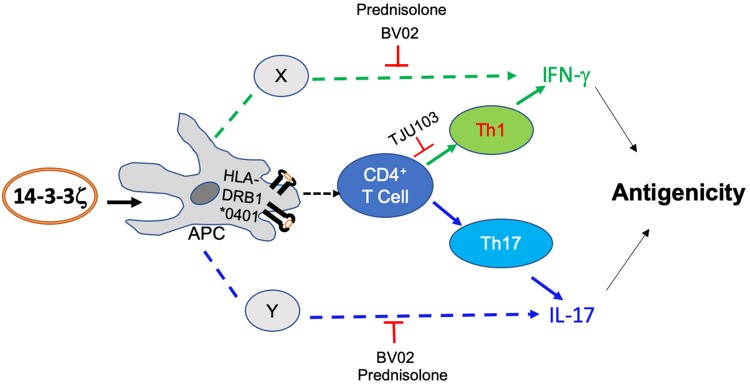
Model depicting 14-3-3ζ-induced antigenic effects. Our results suggest that 14-3-3ζ triggers an increase of IFN-γ and IL-17 in human PBMC by MHC class II-dependent and independent manner, respectively. The result includes the effect on CD4+ cell polarization as well as additional cytokine-producing cells. The effect of 14-3-3ζ on Th1 cells primarily required MHC class II participation. Non-CD4^+^ cells that are capable of producing IFN-γ and IL-17 are more sensitive to 14-3-3 inhibition.

Our study provides a novel insight into the immunological role of 14-3-3ζ that promotes T cell polarization and cytokine production. Using an *ex vivo* human PBMC model, we demonstrated that exogenous 14-3-3ζ triggers IFN-γ and IL-17 production by promoting PBMC proliferation and T cell polarization in favor of Th1 and Th17 cells (Figure 7). Importantly, the T cell function was specific to the 14-3-3ζ isoform and sensitive to immunosuppressive drugs. Prednisolone, an active ingredient of prednisone and commonly prescribed to treat LVV (39), had strong and global effects in suppressing 14-3-3ζ-induced IFN-γ and IL-17 production. Between the Th1 and Th17 polarization, MHC class II presentation of 14-3-3ζ was involved in Th1 cells and IFN-γ production, with no significant impact on Th17 or IL-17 production. The effect of MHC class II was more evident when HLA-DRB1^*^0401 allele presented the 14-3-3ζ to PBMC, which in comparison to another HLA allele had a strong effect on IFN-γ production. In contrast, blocking the adaptor function of 14-3-3 using a competitive inhibitor did not affect T cell polarization, but had a strong effect on the overall cytokine production. Overall, our results show 14-3-3ζ induces IFN-γ and IL-17 cytokine production by promoting T cell polarization and additional mechanisms.

The evidence of antigenic 14-3-3ζ in hundreds of human sera, isolated from healthy individuals and those with a spectrum of diseases, is reported (12, 14, 16, 40). To gain insight into its role, we investigated the immunological functions of 14-3-3ζ. Our results highlight the following key findings: *First*, the exposure to exogenous 14-3-3ζ elicited a response in all tested human PBMC (*n* = 10), which together with the previous reports in human sera suggests that the immune response to 14-3-3ζ is not restricted by the specific MHC presence. On the other hand, the ability of HLA-DRB1^*^0401 to associate and present 14-3-3ζ with strong upregulation of IFN-γ producing cells support its antigenic function. MHC-unrestricted effectors, including T cells, are being recognized as important players of host immune response (41, 42). The presence of multiple immunodominant epitopes, the ability to recognize multiple MHC alleles, and the presence of antigen-specific T cells, can explain MHC-unrestricted antigenicity of 14-3-3ζ. Significant upregulation in IFN-γ producing cells by DRB1^*^0401 presentation of 14-3-3ζ is novel and can be a model to gain further insight into the allele-specific autoimmunity. *Second*, the difference in the immune response to 14-3-3ε suggested that production of IFN-γ and IL-17 is primarily determined by 14-3-3ζ. Since both 14-3-3ζ and 14-3-3ε recombinant proteins were purified using the same protocol and were passed through fresh endotoxin removing columns with 2 h of incubation time (removes >99% endotoxin), it is expected that the immune response is mostly driven by antigenicity of protein, which was confirmed by MHC class II involvement. The specificity is important because 14-3-3ε antigenicity is known in parasitic disease and LVV (20, 43). Recently, the immunosuppressive function of 14-3-3ε by promoting IL-10 based response against *Fasciola gigantica* in goats is reported (24). Though we do not observe the striking influence of 14-3-3ε on the IL-17 or IFN-γ cells, it still can act as a weak antigen, and additional investigations into its immune role are warranted.

*Third*, our results using various immunomodulators suggest that 14-3-3ζ promoted IFN-γ and IL-17 cytokine production included both CD4 and non-CD4 cells. Several non-CD4 cells, including NK, γδT, B, CD8, or neutrophils, are well known to produce IFN-γ and IL-17 (44–46). Additionally, only IFN-γ production by 14-3-3ζ was associated with MHC class II presentation (as tested by TJU103 treatment, HLA blocking antibody, and DRB1^*^0401 expressing cells). Our results suggest that 14-3-3ζ-induced cytokine production requires MHC presentation for IFN-γ and an additional mechanism affecting IL-17. As expected, an immunosuppressive drug such as prednisolone suppressed almost all of 14-3-3ζ antigenic responses. Though the presence of 14-3-3ζ increased the levels of IL-17 and IFN-γ, it had no significant effect on the levels of IL-6, IL-10, or IL-12 during the 7d period tested. Based on cytokine analysis together with the effect on Treg cells, we speculate that Th2 or Tregs are not direct targets of 14-3-3ζ (47–50).

Antigen-stimulated effects on immune cell proliferation and differentiation are gaining the attention of the scientific community (9, 30, 51, 52). However, in addition to the classical antigen presentation processes, several antigens are now recognized as damage-associated molecular pattern (DAMP) molecules that promote IFN-γ and IL-17 levels (53–55). Overall, our results show that 14-3-3ζ is an immunogen that promotes IFN-γ and IL-17 production via MHC presentation and additional mechanisms, respectively. We expect that increased extracellular presence of 14-3-3ζ in the local inflamed tissue can further promote cytokine production. Additionally, identifying the key epitopes and other immune cells that are involved in mediating 14-3-3ζ antigenic effects are important questions for future investigations.

## Materials and Methods

### Reagents

The chemicals were obtained from Sigma-Aldrich and Fisher Scientific Inc. (USA) unless specified. Fluorescently-tagged antibodies for flow cytometry were obtained from Biolegend Inc. ELISA kits for IFN-γ, IL-17, and IL-6 were obtained from Biolegend Inc. ELISPOT plates coated with dual color IFN–γ, IL-17, IL-10, or IL-12, were obtained from Cellular Technology Limited. Polymyxin B based endotoxin removing columns were purchased from Fisher Scientific. The antibody for 14-3-3ζ was purchased from Cell Signaling Technology, the antibody for pan-14-3-3 (clone H-8) was from Santa Cruz Biotechnology, and the antibody for HLA-DR4 was from Sigma.

### Protein Purification

Protein purification was done as mentioned elsewhere (12). Briefly, the BL-21 strain of *E. coli* containing the expression construct of GST-14-3-3ζ (Construct from Addgene) was grown in 10 ml of Luria Broth-Ampicillin (LB-amp) and incubated overnight in a 37°C shaker. Cells were induced with 1 mM IPTG and incubated at 30°C for 1 day at 250 rpm. Bacteria were pelleted and lysed by sonication. Soluble cytosol was then incubated with washed and equilibrated GST resin at 4°C for 4 h. GST-tagged protein was eluted from the washed resin using 10 mM GSH in 100 mM Tris-HCl (pH 8). Eluate was concentrated in a 30 K concentrator before cleaving the GST tag with thrombin (10 unit/mg) for 2 h at 37°C. Cleaved tag was removed by incubating eluate with fresh GST resin for 1.5 h. Final proteins were run through by endotoxin removing spin columns after incubating for at least 2 h in order to achieve maximum endotoxin removal. The purity of the protein was tested by running SDS-PAGE followed by Coomassie staining and western blotting (Supplementary Figure 8).

### PBMC Isolation

Blood from healthy donors was collected as per the approved IRB protocol. Unused banked blood was obtained from the local hospital. The protocol for isolation of PBMCs was adapted from published literature. Briefly, whole blood was diluted with an equal volume of RPMI before layering on Ficoll-Paque PLUS in a 15 mL tube. Tubes were then centrifuged for 25 min with the brakes off at 950 g. PBMCs were collected from the layer between the plasma and Ficoll liquid. PBMCs were then washed twice with RPMI, with 7 min spins at 350 g to pellet the cells. Finally, cells were resuspended in complete RPMI before counting and plating.

### PBMC Assays

Cells were plated with 14-3-3ζ protein in a 96-well plate for 0–8 days at 37°C in complete RPMI culture medium. Phytohemagglutinin (PHA) at 2% or other proteins at 1 μg/ml were used as controls. For viability, 10 μl MTT (5 mg/ml) was added to each well, and plate was further incubated at 37°C in the dark for 3 h. The supernatant was then removed, and 100 μL DMSO was added for another hour before reading the absorbance at 570 nm using FLUOstar Omega microplate reader (BMG Labtech). For PI staining, PBMC were stained with 10 μl of dye per million cells for 15 min at 4°C in the dark. For T cell polarization assay, PBMC were incubated with 1 μg/ml of 14-3-3ζ for 7d followed by either the cellular staining for FACS analysis or ELISPOT. For testing the effect of drugs or inhibitors, we added prednisolone, prednisone as well as TJU103 at 1 μg/ml, BV02 at 5 nM, and antibodies at 2 μg/ml for 30 min before the addition of 14-3-3ζ. Drugs were supplemented every 48 h during the 7d period. The antigenic presentation in DRB1^*^0401 expressing cells was carried out by incubating 50,000 cells with 1 μg/ml of 14-3-3ζ for 24 h in RPMI media supplemented with non-essential amino acids, followed by washing cells twice with the media to remove unprocessed antigen. Washed cells were then added to 500,000 PBMC and co-cultured for 7d followed by ELISPOT.

### ELISPOT Assay

The protocol was adopted from the Immunospot kit from Cellular Technology Limited. Briefly, PBMC incubated with 14-3-3ζ for 7 d were plated on the antibody-coated ELISPOT plates. In each well, 100,000 cells were added and incubated for 2 d at 37°C, followed by the recommended developing protocol. Plates were read with a Quantihub ELISPOT plate reader, and spots were counted manually. IFN-γ and IL-10 spots showed up as red-colored, and IL-17A and IL-12 spots showed up as blue colored. The results are represented as number of spots per million cells.

### Flow Cytometry

PBMC incubated with 14-3-3ζ for 7 d at 37°C were fixed in 2% paraformaldehyde for an hour and washed with PBS. Cells for IL-17 staining were treated with 10 μg/mL brefeldin for two hours before the fixation. For surface staining, the cells were immunostained in 50 μL PBS with 5 μl antibody per million cells for 30 min in the dark on ice. For intracellular staining (IL-17, Foxp3), the cells were permeabilized with Triton-X100 for 15 min. After staining, the cells were washed with PBS and resuspended in 1 mL PBS for analysis. Following antibodies from BioLegend Inc. were used: Cy5 or FITC-conjugated CD3 (clone HIT3a), FITC-conjugated anti-human CD4 (clone A161A1), APC-conjugated anti-human CD366 (clone F38-2E2) and Alexa Fluor 647-conjugated anti-human IL-17A (clone BL168). Cells were scanned at the FACSCanto II system (BD Biosciences) and analyzed using FlowJo software (version 10). A gate for live cells was created around an initial population of cells in the FSC-SSC plot, and this population of cells was further analyzed for CD3, CD4, CD366, and IL-17 expression. Unstained and isotype labeled cells were used as a control for setting the gates.

### ELISA

Cytokine levels in the conditioned medium were measured using the commercial kits as per protocols provided by the manufacturer (BioLegend Inc).

### Cell Culture

Cells co-expressing HLA-DRB1^*^0401 and DM (a kind gift from Dr. Anthony W. Purcell at Monash University, Australia), were cultured in RPMI media containing 10% FBS and non-essential amino acids.

### Statistical Analysis

All data were plotted using GraphPad (La Jolla, CA). Data is indicated as mean with standard error. Two-tailed, unpaired student's *t*-test was used to determine the significance of the difference between the two groups. Statistical significance was set at the value of <0.05. All other comparisons were analyzed by one-way analysis of variance (ANOVA) with Bonferroni's multiple-comparison analysis, and statistical significance was set at a *P*-value of <0.05.

## Data Availability

The raw data supporting the conclusions of this manuscript will be made available by the authors, without undue reservation, to any qualified researcher.

## Ethics Statement

This study was carried out in accordance with the recommendations of the institutional committee with written informed consent from all subjects. All subjects gave written informed consent in accordance with the Declaration of Helsinki. The protocol was approved by the University of Toledo IRB 201986.

## Author Contributions

RC and SC designed the experiment. JM, CP, and RC executed the experiment. RC arranged administrative and financial support for the project.

### Conflict of Interest Statement

The authors declare that the research was conducted in the absence of any commercial or financial relationships that could be construed as a potential conflict of interest.
