# Supplementary material for: 14-3-3ζ-A Novel Immunogen Promotes Inflammatory Cytokine Production
Source: Front Immunol. 2019 Jul 24;10:1553. doi: 10.3389/fimmu.2019.01553 (PMC6667649; doi:10.3389/fimmu.2019.01553)
Supplement: Supplementary file 1 [file Data_Sheet_1.pdf]

14-3-3ζ Antigenicity Promotes Inflammatory Cytokine Production- Supplementary Data- Revision

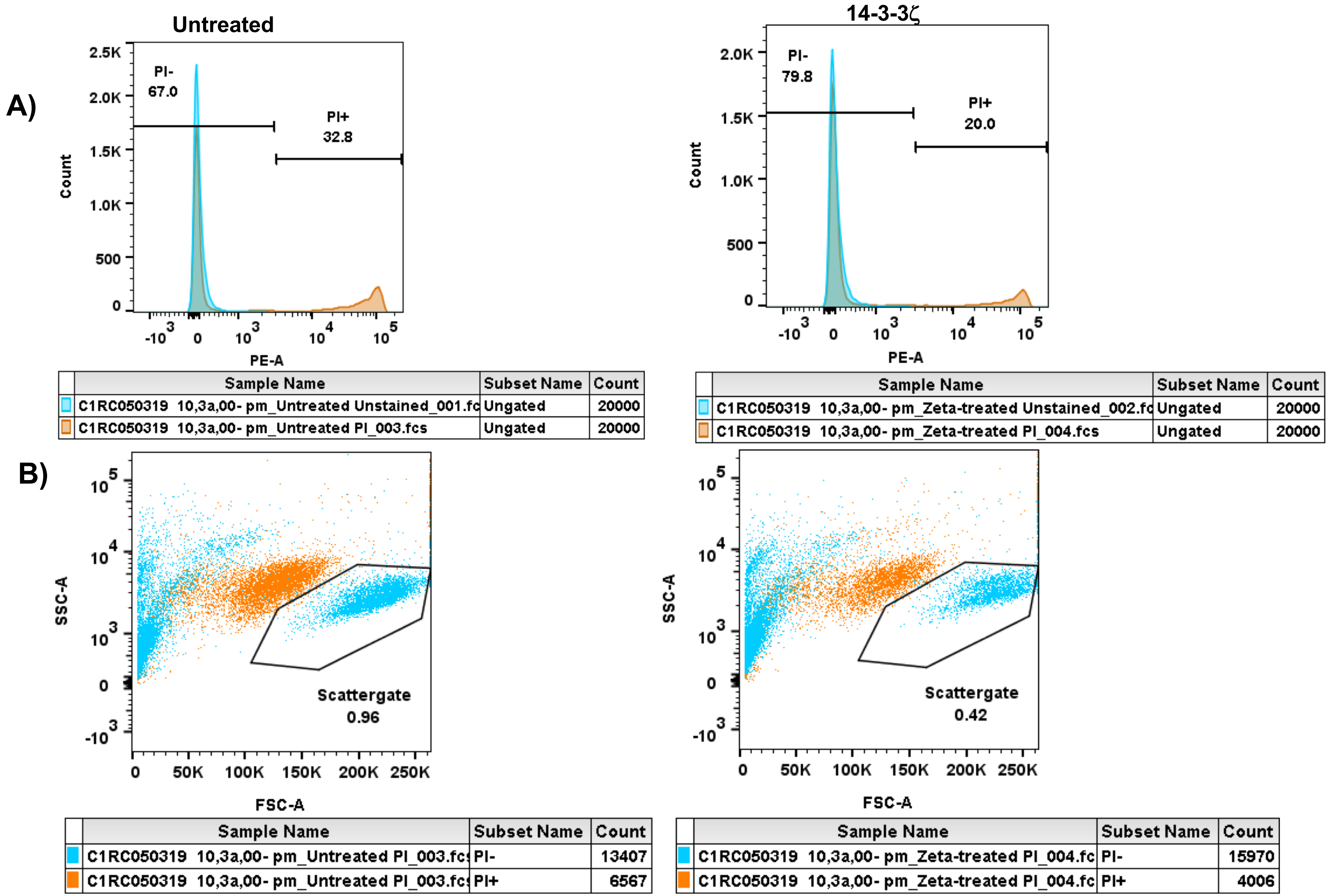

Supplementary Figure 1: PBMC after 7d in culture with or without the 14-3-3ζ treatment were stained with propidium iodide (PI). (A) Representative histograms showing PI-stained (orange) and unstained (blue) gates were determined. (B) Forward scatter vs side scatter plots show that scattergate excludes PI stained dead cells (orange) from our analyses. (n=2)

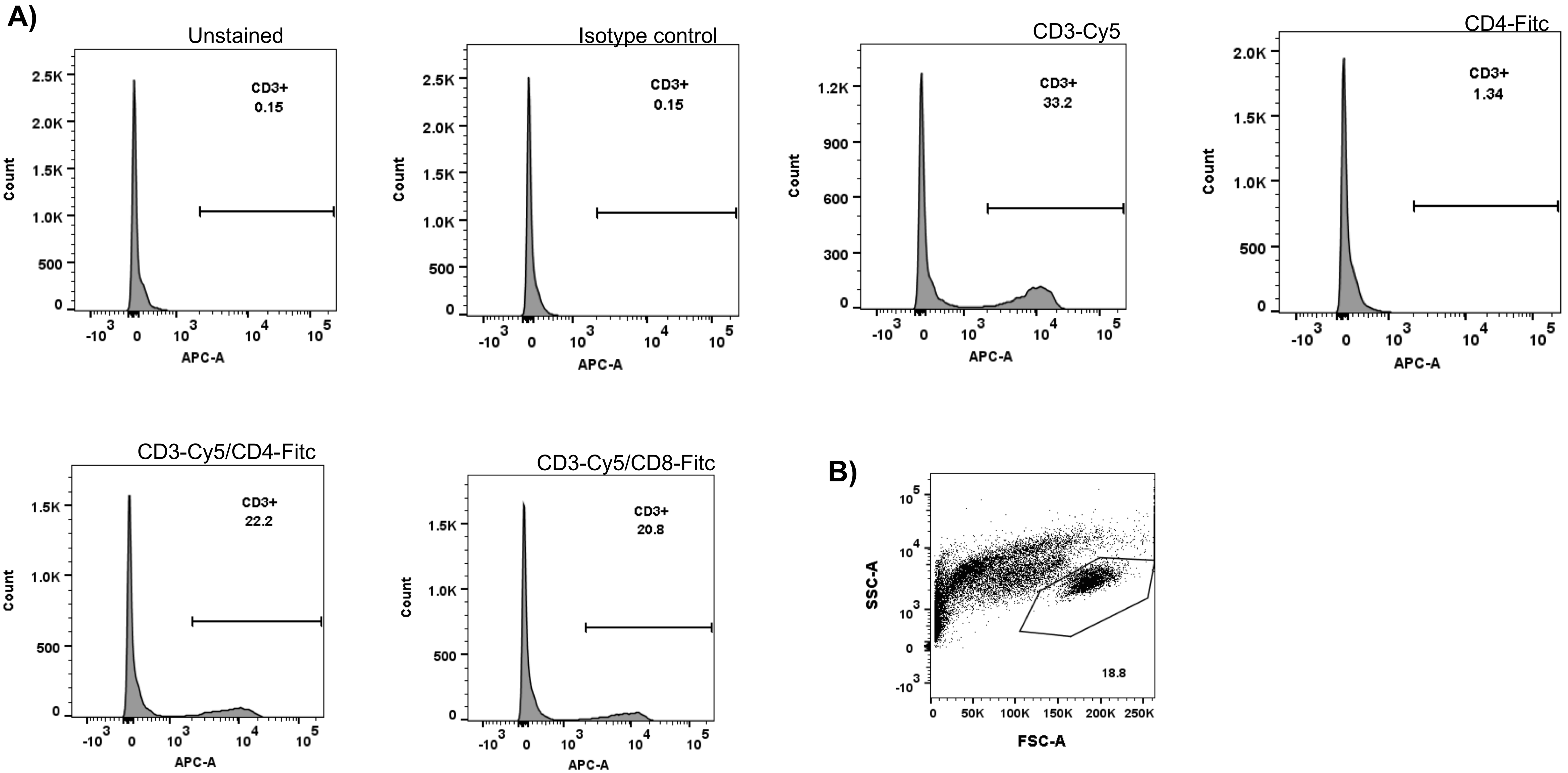

Supplementary Figure 2: (A) Representative CD3+ gate is shown for the PBMC treated with 14-3-3ζ for 7d in culture. Cells were stained CD3 (Cy5), CD4 (Fitc) and CD8 (FITC) alone or in combinations. (B) A representative gate based on the forward and side scatter together with live CD4 cells used to investigate 14-3-3ζ effect on T cells is shown.

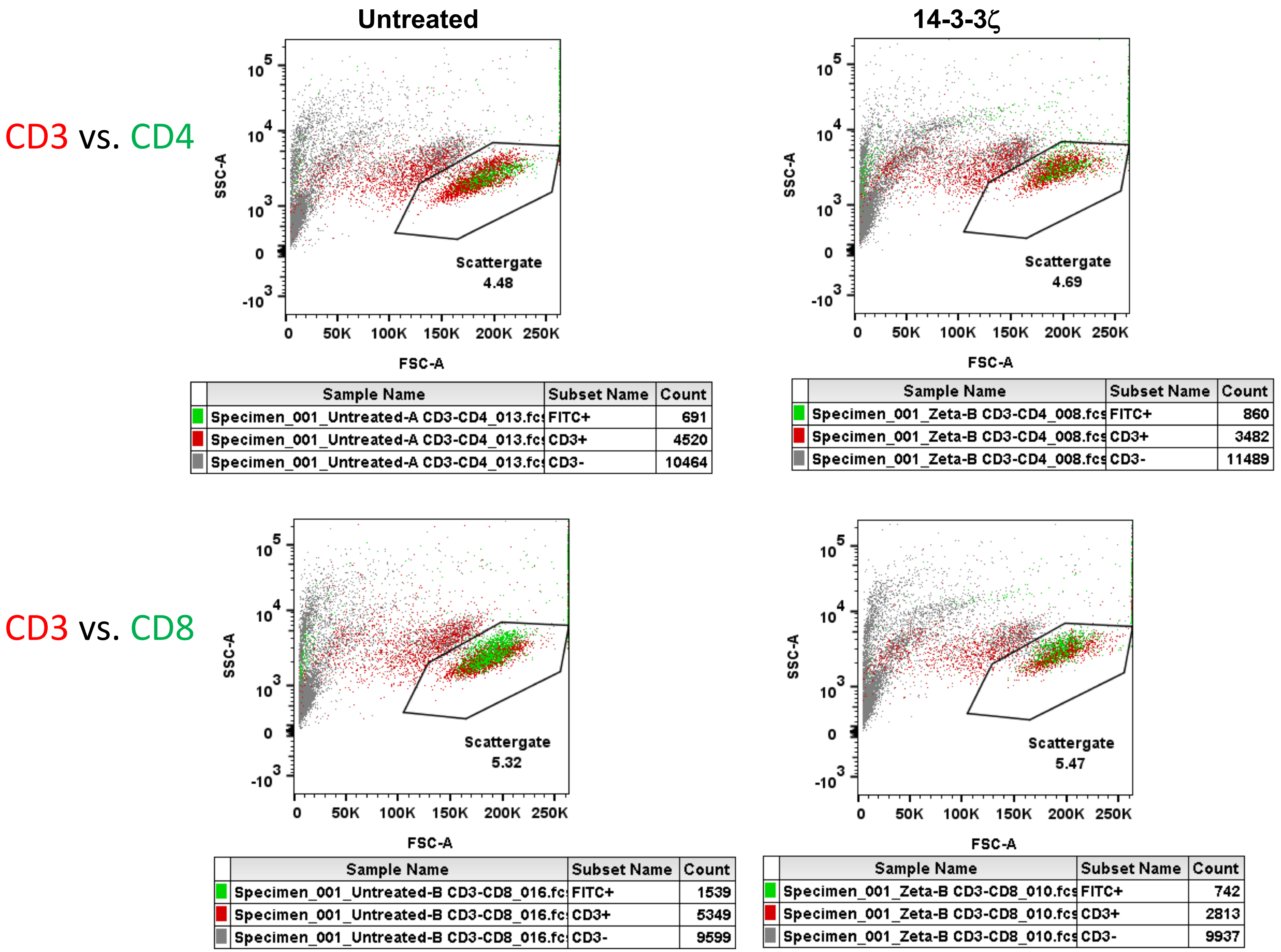

	Sample Name	Subset Name	Count
green	Specimen_001_Untreated-A CD3-CD4_013.fc	FITC+	691
red	Specimen_001_Untreated-A CD3-CD4_013.fc	CD3+	4520
grey	Specimen_001_Untreated-A CD3-CD4_013.fc	CD3-	10464

	Sample Name	Subset Name	Count
green	Specimen_001_Zeta-B CD3-CD4_008.fcs	FITC+	860
red	Specimen_001_Zeta-B CD3-CD4_008.fcs	CD3+	3482
grey	Specimen_001_Zeta-B CD3-CD4_008.fcs	CD3-	11489

	Sample Name	Subset Name	Count
green	Specimen_001_Untreated-B CD3-CD8_016.fc	FITC+	1539
red	Specimen_001_Untreated-B CD3-CD8_016.fc	CD3+	5349
grey	Specimen_001_Untreated-B CD3-CD8_016.fc	CD3-	9599

	Sample Name	Subset Name	Count
green	Specimen_001_Zeta-B CD3-CD8_010.fcs	FITC+	742
red	Specimen_001_Zeta-B CD3-CD8_010.fcs	CD3+	2813
grey	Specimen_001_Zeta-B CD3-CD8_010.fcs	CD3-	9937

Supplementary Figure 3: Selection of lymphocyte gate based on CD3 staining is shown. Representative CD3-PEcy5 (shown as red), CD4-FITC (shown as green) and CD3-PEcy5 (shown as red), CD8-FITC (shown as green) stained cells for untreated and 14-3-3ζ–treated PBMC are shown. CD3-PEcy5-negative cells are shown in grey.

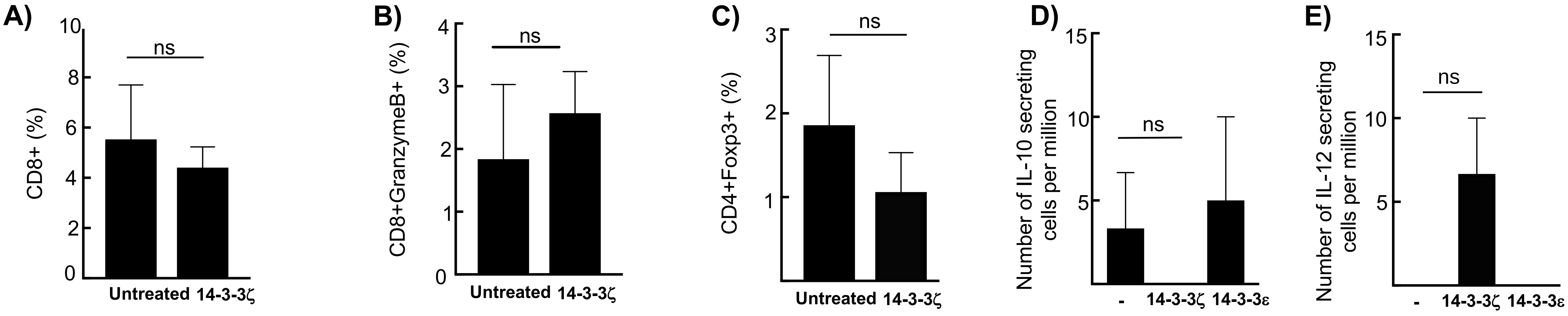

Supplementary Figure 4- The effect of 14-3-3ζ on the total number of CD8 cells (A), Granzyme B positive CD8 cells (B), FOXP3 positive CD4 cells (C), number of IL-10 secreting cells (D), and number of IL-12 secreting cells (E) was analyzed. (n >2)

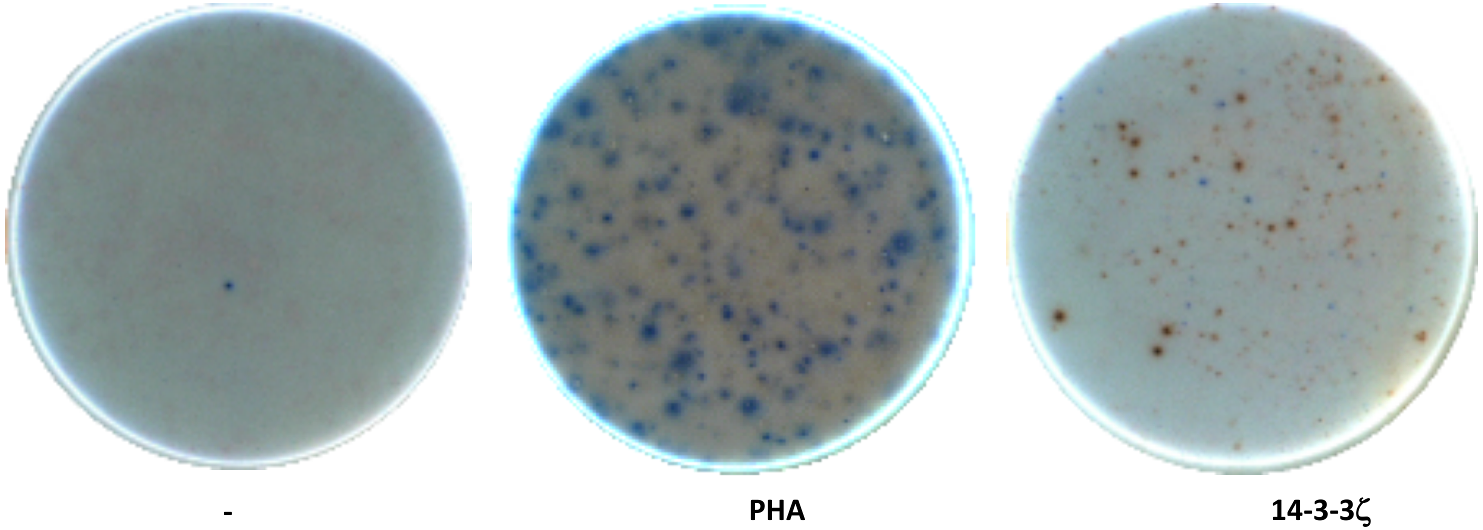

Supplementary Figure 5- Representative wells of ELISPOT assay showing IFN-γ and IL-17 secreting cells as red and blue, respectively.

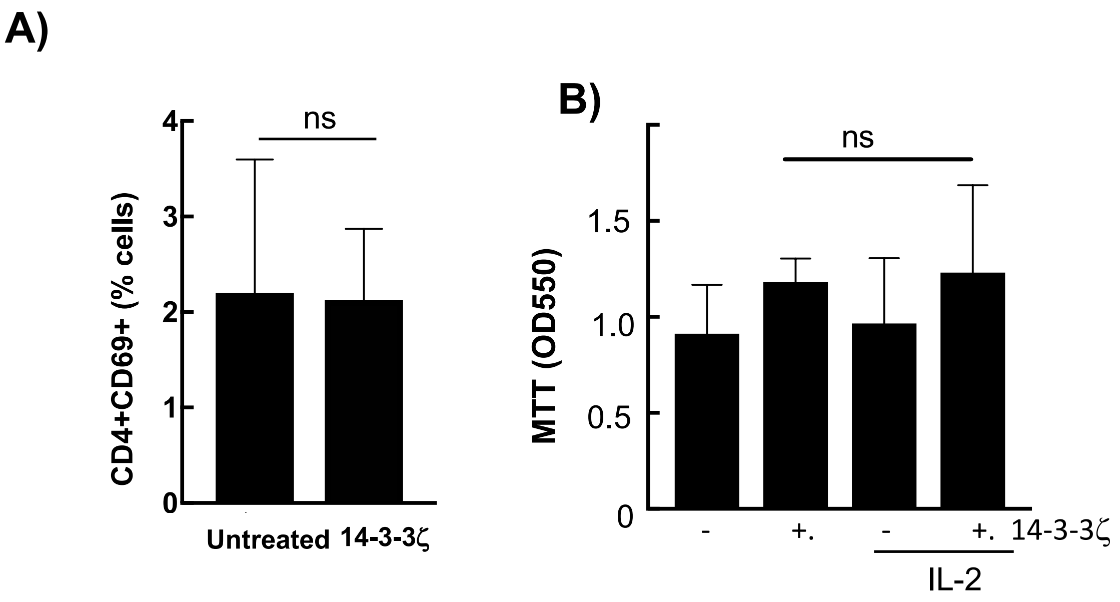

Supplementary Figure 6- (A) The effect of 3d incubation of 14-3-3ζ on CD69 expression was analyzed. (B) The effect of IL-2 on PBMC proliferation was analyzed using MTT assay (n=2).

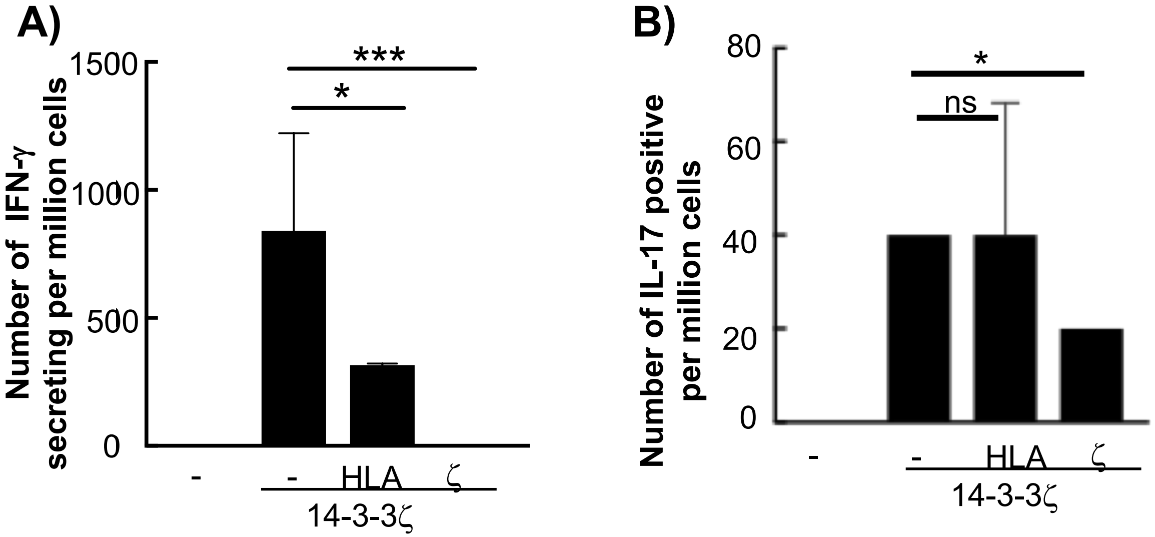

Supplementary Figure 7- HLA-DR4 blocking suppresses 14-3-3ζ-induced IFN-γ production. (A-B) Effect of treating PBMC with HLA antibody (clone MEM136) or 14-3-3 antibody (clone H8, SCBT) before the antigen addition was measured on IFN-γ (A) and IL-17 (B) secreting cells by ELISPOT assay (n=2).

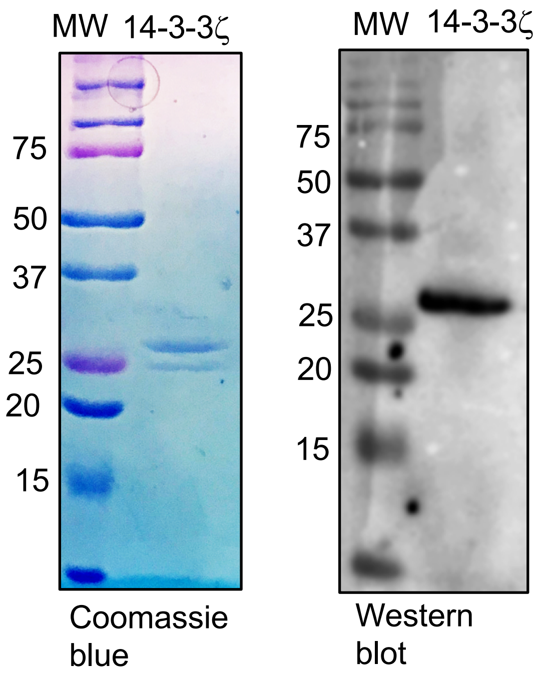

Supplementary Figure 8- Purity of recombinant 14-3-3ζ purified in the coomassie stain and western blot is shown

% Positive Cell	Scatter gate based on CD3		Scatter gate based on lymphocyte gate (CD3 positive live cells)	
	Untreated-7d	14-3-3ζ treated-7d	Untreated-7d	14-3-3ζ treated-7d
CD3+	47±1.6	34 ± 0.8	38.05 ±0.85	25.57 ± 0.235
CD3+CD4+	13.65 ±2.75	9.85 ± 1.45	3.8. ±0.8	4.39 ±1.34
CD3+CD8+	11.33 ±1.77	6.0 ±0.1	8.175 ±1.125	4.87 ± 0.07

Supplementary Table 1: Calculations of CD3, CD4 and CD8 cells for the PBMC (n=2) treated with 14-3-3ζ for 7d using the CD3 gate (Supplementary Fig 2A) or lymphocyte gate (Supplementary Fig 2B) are shown.
